# Clinico-Pathological Features and Prognosis of Invasive Micropapillary Carcinoma Compared to Invasive Ductal Carcinoma: A Population-Based Study from China

**DOI:** 10.1371/journal.pone.0101390

**Published:** 2014-06-30

**Authors:** Wen-Biao Shi, Lin-Jun Yang, Xin Hu, Jian Zhou, Qiang Zhang, Zhi-Ming Shao

**Affiliations:** 1 Department of Breast Surgery, Key Laboratory of Breast Cancer in Shanghai, Fudan University Shanghai Cancer Center, Shanghai, China; 2 Department of Oncology, Shanghai Medical College, Fudan University, Shanghai, China; 3 Department of Surgical Oncology, Taizhou Municipal Hospital, Taizhou, Zhejiang, China; University of North Carolina School of Medicine, United States of America

## Abstract

**Background:**

Invasive micropapillary carcinoma (IMPC) of the breast is a rare subtype of breast cancer that is associated with a high incidence of regional lymph node metastases and a poor clinical outcome. However, the clinico-pathological features and prognostic factors of IMPC are not well understood.

**Patients and Methods:**

A total of 188 IMPC cases and 1,289 invasive ductal carcinoma (IDC) cases were included. The clinical features, breast cancer-specific survival (BCSS) and recurrence/metastasis-free survival (RFS) of the patients were compared between these two groups.

**Results:**

The IMPC patients exhibited more features of aggressive carcinoma than the IDC patients, including larger tumor size, higher tumor stage, a greater proportion of nodal involvement and an increased incidence of lymphovascular invasion. Patients with IMPC had lower 5-year BCSS and RFS rates (75.9% and 67.1%, respectively) than patients with IDC (89.5% and 84.5%, respectively). Compared to IDC patients, the patients with IMPC had a significantly higher percentage of stage III breast cancer (51.3% versus 21.7%). In a stage-matched Kaplan-Meier analysis, the patients with stage III IMPC had lower 5-year BCSS and RFS rates than patients with stage III IDC (BCSS, P = 0.004; RFS, P = 0.034). A multivariate analysis revealed that TNM stage was an independent prognostic factor for patients with IMPC. The proportion of cancers with a luminal-like subtype was significantly higher in IMPC than in IDC (P<0.001). However, after matching by molecular subtype, the patients with IMPC had significantly worse clinical outcomes than patients with IDC.

**Conclusions:**

In Chinese women, IMPCs displayed more aggressive behaviors than IDCs, resulting in poorer clinical outcomes for patients with IMPC, regardless of a favorable molecular subtype. Our findings illustrate that the poorer survival of patients with IMPC might be due to an increased incidence and aggressiveness of tumors in TNM stage III.

## Introduction

Invasive micropapillary carcinoma (IMPC) of the breast is an uncommon and distinct variant of breast cancer that is characterized by pseudopapillary and tubuloalveolar arrangements of tumor cell clusters in sponge-like, clear empty spaces, thereby mimicking extensive lymphatic invasion [1]. This carcinoma has been reported to exhibit lymphovascular invasion, lymph node metastasis, local recurrence and distant metastasis at relatively high frequencies, thus exhibiting a more aggressive behavior than invasive ductal carcinoma (IDC) [2], [3]. The rate of incidence of IMPC of the breast has been reported to range from 1.0–8.4% [4], [5], [6], [7], [8], [9], [10]. Due to the low incidence of this breast cancer variant, most studies examining IMPC have small sample sizes; the clinico-pathological characteristics and the clinical prognostic factors of invasive micropapillary carcinoma are therefore not well understood. It is worth noting that the molecular subtypes of breast carcinomas have been extensively studied and demonstrated to have significant clinical value [11], [12]. However, to our knowledge, there is limited information available that is specifically related to the IMPC molecular subtype.

Therefore, we conducted an extensive comparison study of IMPC and IDC patients in a large-scale cohort to provide a more complete and reliable overview of the clinico-pathological features and prognostic factors of IMPC.

## Methods

### Patients and Follow-up

We retrospectively reviewed the data of 188 consecutive patients with IMPC who were diagnosed histopathologically and treated at the Department of Breast Surgery of the Fudan University Shanghai Cancer Center (FUSCC) from January 2007 to October 2012. All IMPC cases included in the study displayed a micropapillary tumor component that was in accordance with the morphological criteria described in the WHO histological classification of tumors of the breast [13]. As the number of IMPC cases was relatively small, an equally small number of IDC controls would provide little ability to find associations. Increasing the number of controls to a ratio greater than 4/1 would improve the power of the study [14]. Therefore, based on the number of IMPC patients enrolled during each year of the study period, approximately 7-fold patients with IDC were selected via a simple random sampling method from the corresponding year; a total of 1,289 of the recruited IDC cases were enrolled as control patients. Tumors were histologically classified as IDC according to the WHO classification criteria. IDC cases that were mixed with the IMPC component were excluded from the control IDC group. Of the 188 IMPC cases, 27 patients (14.4%) were identified as having pure IMPC, whereas 161 patients (85.6%) had mixed IMPC (Table S1). The nonmicropapillary invasive carcinoma components of the mixed IMPC cases were as follows: IDC, mucinous carcinoma, and ductal carcinoma in situ. The histological grade, Ki-67 index and the proportion of the IMPC component of each mixed IMPC specimen were not analyzed due to a lack of available information in many cases. All patients were female and had no distant metastasis at the time of their primary diagnosis. The pathological tumor stage (TNM stage) was assessed according to the criteria established by the 6th edition of the American Joint Committee on Cancer (AJCC) staging manual. Patients were evaluated according to clinical practice guideline standards with a complete physical examination, chest X-ray, ECG, complete blood count, routine biochemical tests, and bilateral mammography; the patients also received ultrasonography of the breasts, axillary fossae, cervical region, abdomen, and pelvis prior to surgery and the accompanying adjuvant therapy. This study was approved by the independent ethical committee/institutional review board of FUSCC (Shanghai Cancer Center Ethical Committee), and all patients provided written informed consent.

Follow-up information regarding tumor relapse and survival status was available through outpatient departmental records and personal contact with the patients via mail and telephone calls. The follow-ups were carried out at FUSCC every 3 months during the first 2 years, every 6 months during the next 2 years and once a year thereafter.

### Pathology Analysis

The status of the estrogen receptor (ER), progesterone receptor (PR), and human epidermal growth factor receptor-2 (HER-2) were determined by immunohistochemical (IHC) staining, which was performed using a standard operating procedure in the department of pathology of FUSCC. The cut-off value for ER positivity and PR positivity was 1% of tumor cells with positive nuclear staining. Tumors with an IHC score of 3+ based on circumferential membrane-bound staining (DakoCytomation, Carpinteria, CA, US) or with amplification confirmed by florescent in situ hybridization were defined as HER-2-positive. In this study, we categorized the breast tumors into four groups as follows: luminal A (ER and/or PR positive, HER-2 negative), luminal B (ER and/or PR positive, HER-2 positive), HER-2+ (ER and PR negative, HER-2 positive) and triple negative (TN) (ER negative, PR negative, and HER-2 negative), according to the standard described by Carey [15].

### Statistical Analysis

Breast cancer-specific survival (BCSS) was defined as the interval between diagnosis and death due to breast cancer progression. Recurrence/metastasis-free survival (RFS) was defined as the interval from the same start date to the date of disease relapse (local, regional, or distant). Patients who died of other causes were censored at the date of their death for BCSS analysis. Those without any evidence of relapse were censored at the last date on which they were known to be alive. The clinico-pathological parameters of the different subgroups were compared using an independent sample t-tests and Pearson's chi-square test; Fisher's exact test was used when needed. The survival rates were estimated using the Kaplan-Meier method and compared using the log-rank test. Univariate and multivariate analyses were performed using the Cox risk proportion model, and all variables in the univariate analysis were investigated by multivariate analysis. Hazard ratios (HR) were presented with 95% confidence intervals. All statistical tests were two sided, and P<0.05 was considered to be significant. The SPSS 17.0 software package (SPSS, Chicago, IL, USA) was used for statistical analysis.

## Results

### The clinico-pathological characteristics of patients with IMPCs and IDCs

The clinical and pathological characteristics of the patients included in the study are shown in Table 1. A total of 188 patients were diagnosed with IMPC; the median age of these patients was 52.7 years, and the range was from 30 to 87 years. The IDC group included 1,289 patients: the median age of these patients was 51.7 years, and the range was from 21 to 91 years. The percentage of large tumors (greater than 2 cm in diameter) was higher in the IMPC patients than in the IDC patients (65.2% versus 51.7%, P = 0.002). Furthermore, the patients with IMPC were more likely to have a positive node status (P<0.001) and had a significantly higher incidence of lymphovascular invasion (75.4% for IMPCs and 36.5% for IDCs, P<0.001). The IMPC cases also displayed a tight association with higher breast cancer TNM stage (TNM stage III, P<0.001).

**Table 1:** Baseline characteristics and treatment patterns for all patients.

	IMPC (n = 188)		IDC (n = 1289)		
Characteristics	n	%	n	%	*P* value
**Age, year (Mean±SD)**	52.7±11.3		51.7±10.4		0.196
**Menopausal status**					0.480
Premenopausal	81	43.1	582	46.0	
Postmenopausal	107	56.9	682	54.0	
Unknown			25		
**Tumor size, cm**					**0.002**
T≤2	65	34.8	609	48.3	
2<T≤5	104	55.6	567	45.0	
T>5	18	9.6	84	6.7	
Unknown	1		29		
**Node status**					**<0.001**
0	50	26.6	702	55.3	
1–3	51	27.1	310	24.4	
4–9	48	25.5	158	12.5	
≥10	39	20.7	99	7.8	
Unknown			20		
**TNM stage**					**<0.001**
I	27	14.4	396	31.4	
II	64	34.2	593	47.0	
III	96	51.3	274	21.7	
Unknown	1		26		
**ER status**					**<0.001**
Positive	160	85.1	908	72.5	
Negative	28	14.9	345	27.5	
Unknown			36		
**PR status**					**<0.001**
Positive	147	78.2	864	69.0	
Negative	41	21.8	389	31.0	
Unknown			36		
**HER2 status**					0.122
Positive	55	29.9	307	24.5	
Negative	129	70.1	946	75.5	
Unknown	4		36		
**Lymphovascular invasion**					**<0.001**
Yes	135	75.4	405	36.5	
No	44	24.6	706	63.5	
Unknown	9		178		
**Subtype**					**<0.001**
Luminal	163	88.6	972	77.6	
Non-luminal	21	11.4	281	22.4	
Unknown	4		36		
**Surgery**					**<0.001**
Mastectomy	179	95.2	1088	84.4	
BCS	9	4.8	201	15.6	
**Neoadjuvant chemotherapy**					**0.026**
Yes	56	29.8	287	22.3	
No	132	70.2	1002	77.7	
**Adjuvant chemotherapy**					0.656
Yes	176	93.6	1162	92.4	
No	12	6.4	96	7.6	
Unknown			31		
**Adjuvant radiotherapy**					**0.005**
Yes	103	57.5	572	46.3	
No	76	42.5	663	53.7	
Unknown	9		50		
**Adjuvant endocrine therapy**					**0.023**
Yes	154	87.5	1016	80.4	
No	22	12.5	248	19.6	
Unknown	12		25		

**Abbreviations**: IMPC, invasive micropapillary carcinoma; IDC, invasive ductal carcinoma; TNM, tumor, node, metastasis; ER, estrogen receptor; PR, progesterone receptor; HER-2, human epidermal growth factor receptor 2; BCS, breast-conserving surgery;

**P-value is calculated by two-sided χ2 test;**

**Bold values denote P<0.05.**

ER status was positive in 85.1% of IMPCs and 72.5% of IDCs (P<0.001). The proportion of PR-positive tumors was 78.2% for IMPCs and 69.0% for IDCs (P = 0.010). No significant difference was observed between the two groups regarding the status of HER-2 (P = 0.122). Human breast tumors can be classified into luminal A, luminal B, HER-2+, and triple-negative (TN) subtypes based on the expression status of ER, PR, and HER-2. In IMPC, 67.9% of the samples were of the luminal A subtype, 20.7% of the samples were of the luminal B subtype, 2.2% were of the HER-2+ subtype, and 9.2% were of the TN subtype. Among the IDC samples, 63.4% were of the luminal A subtype, 14.2% were of the luminal B subtype, 12.1% were of the HER-2+ subtype, and 10.3% were of the TN subtype. Due to the low incidence of the HER-2+ and TN subtypes, we combined these two subtypes into a single non-luminal subtype and combined luminal A and luminal B into a single luminal subtype. Using this classification, we observed that the proportion of tumors with a luminal subtype was significantly higher in IMPCs than in IDCs (88.6% for IMPCs and 77.6% for IDCs, P<0.001).

The surgical management of the breast cancer patients is summarized in Table 1. At the initial assessment, all of the patients were offered mastectomy or breast-conserving surgery (BCS) in accordance with the National Comprehensive Cancer Network (NCCN) guidelines. In our study, the patients with IMPC underwent BCS at a lower frequency than the patients with IDC. A higher proportion of IMPC cases received adjuvant endocrine therapy (P = 0.023), and no significant difference in the frequency of adjuvant chemotherapy administration was observed (P = 0.656). Relative to those with IDC, patients with IMPC were more likely to receive neoadjuvant chemotherapy and adjuvant radiotherapy (P = 0.026 and P = 0.005, respectively), most likely because these patients were more likely to have late-stage disease.

### A comparative Kaplan-Meier survival analysis of IMPCs and IDCs

**Figure 1:**
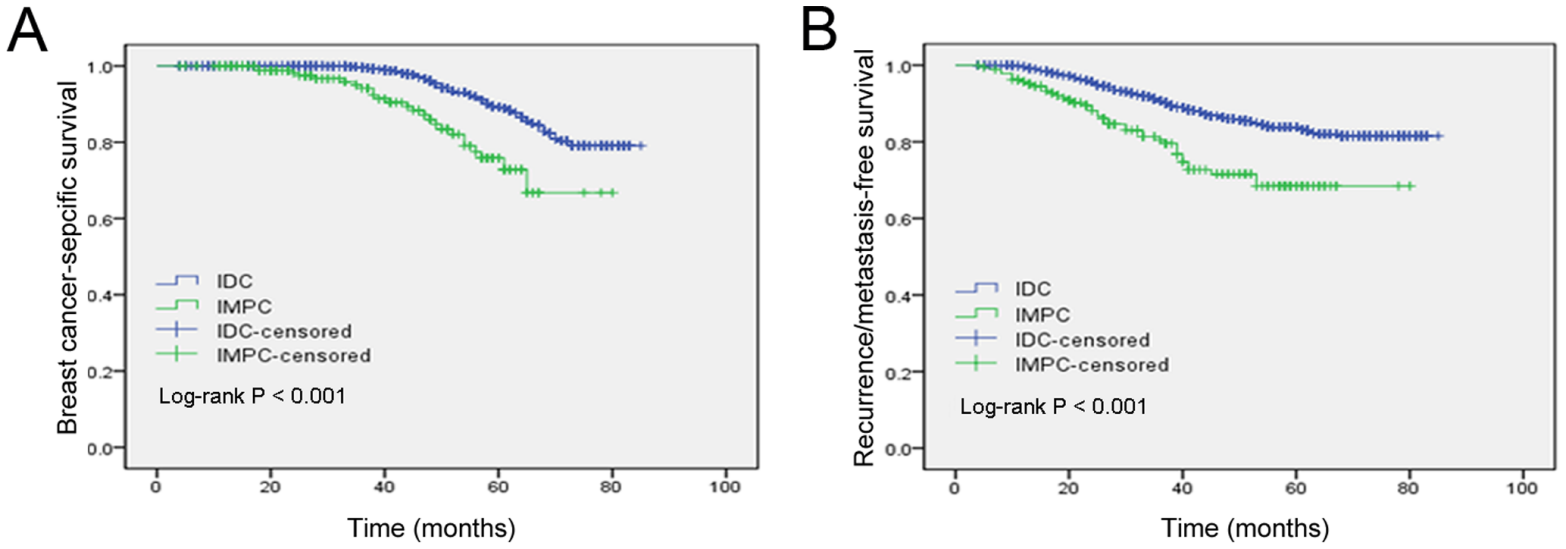
Kaplan-Meier estimates of breast cancer-specific survival (A) and recurrence/metastasis-free survival (B) according to the histologic types in the general population.

**Figure 2:**
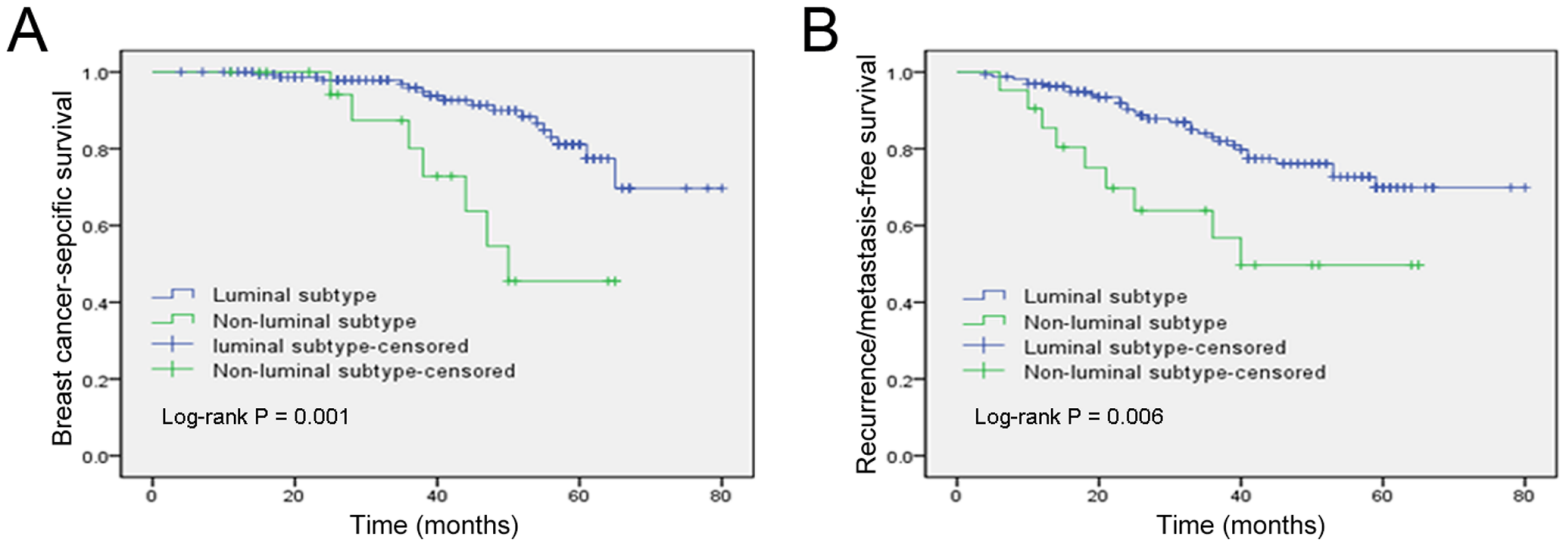
Kaplan-Meier estimates of breast cancer-specific survival (A) and recurrence/metastasis-free survival (B) according to the molecular subtype in the IMPC group.

The median follow-up of all patients was 48 months (40.5 months for the IMPC group vs. 50 months for the IDC group), with a range of 4 to 83 months. A total of 73 patients were lost during the follow-up period. In our cohort, the women diagnosed with IMPC had a higher frequency of recurrence or metastasis (P<0.001) and death (P<0.001) compared to those with IDC. As shown in Figure 1, the Kaplan-Meier analysis revealed that patients with IMPC had poorer 5-year BCSS and RFS rates (75.9 and 67.1%, respectively) than patients with IDC (89.5 and 84.5%, respectively). The stratification of breast tumors by molecular subtype indicated that patients with luminal-subtype tumors had significantly better BCSS and RFS times than patients with non-luminal subtypes (Figure 2).

**Figure 3:**
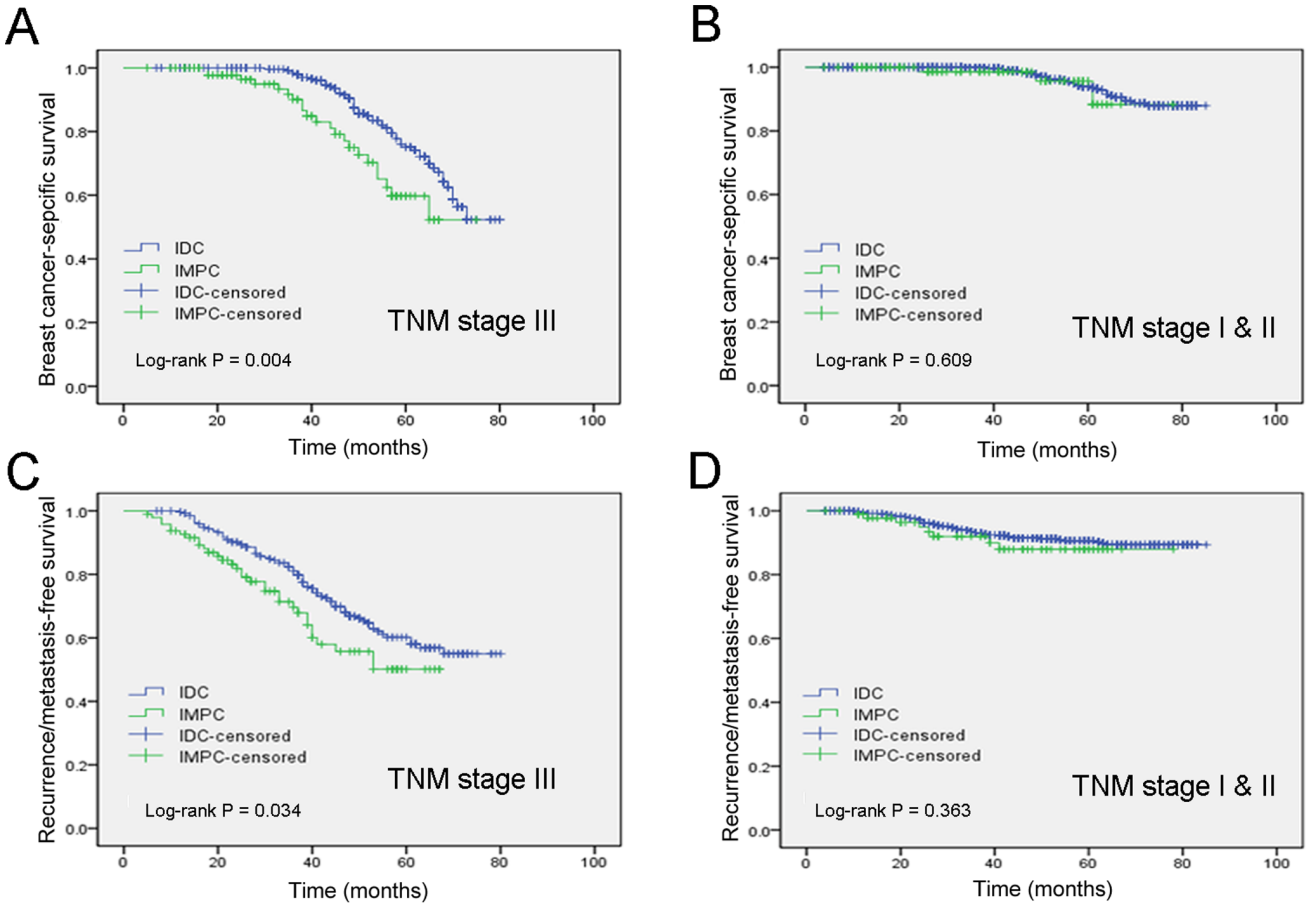
Kaplan-Meier estimates of breast cancer-specific survival in TNM stages III (A) and I & II (B) and recurrence/metastasis-free survival in stages III (C) and I & II (D) according to the histologic type in the general population.

**Figure 4:**
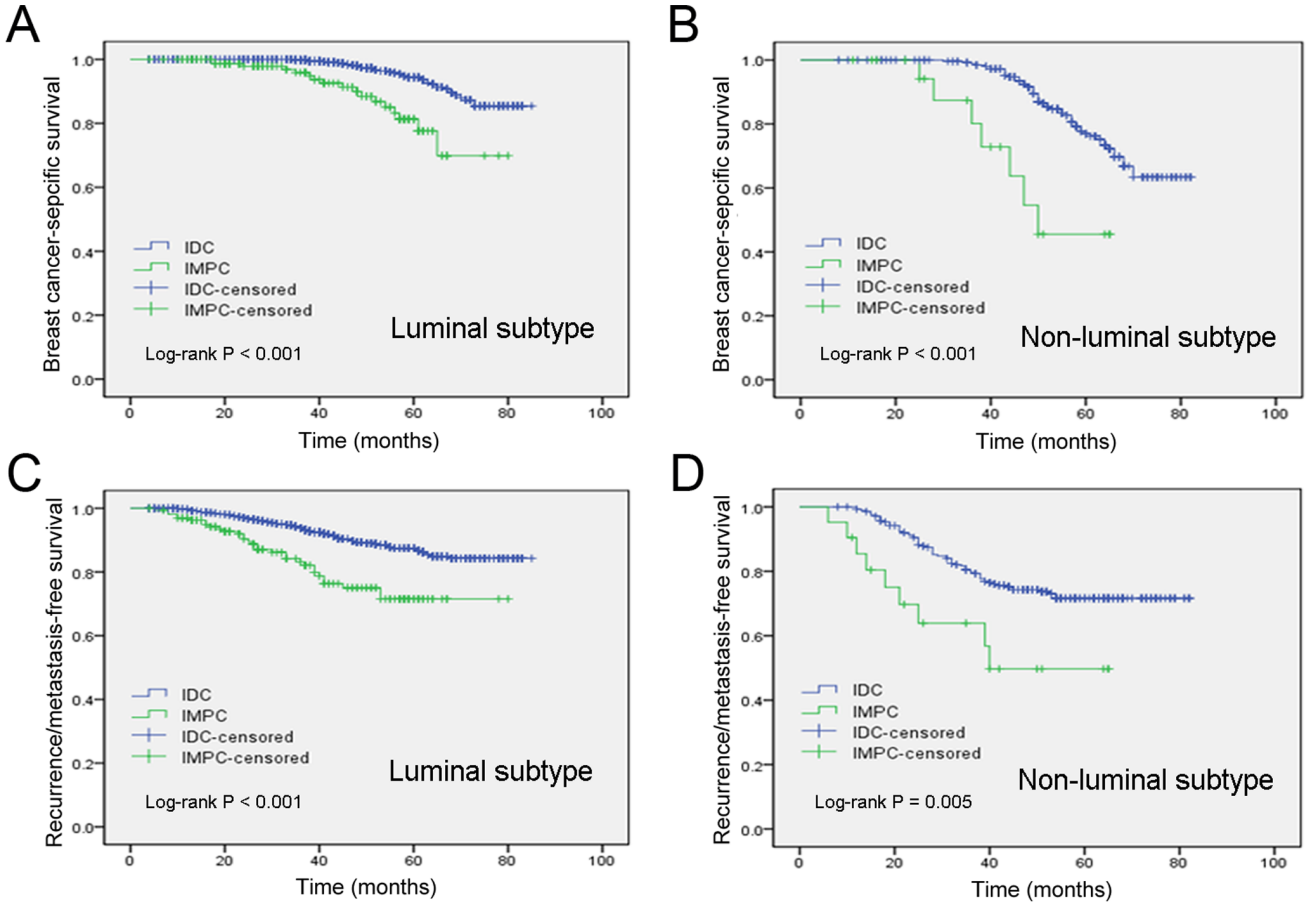
Kaplan–Meier estimates of breast cancer-specific survival in the luminal subtypes (A) and the non-luminal subtypes (B) and recurrence/metastasis-free survival in the luminal subtypes (C) and the non-luminal subtypes (D) according to histologic type in the general population.

As shown in Table 1, patients with IMPC had a significantly higher rate of stage III breast cancer than patients with IDC (stage III, 51.3% versus 21.7%); in contrast, the percentage of stage I or stage II breast cancer was lower in the IMPC patients than in the IDC patients (stage I, 14.4% versus 31.4%; stage II, 34.2% versus 47.0%). Therefore, we divided the patients into two subgroups (stage I & II and stage III). In a stage-matched analysis of BCSS and RFS, patients with stage III IMPC had poorer 5-year BCSS and RFS than patients with stage III IDC (BCSS, P = 0.004 and RFS, P = 0.034; Figure 3A and 3C). However, there were no statistically significant differences in the 5-year BCSS rate and RFS rate between the IMPC group and the IDC group after matching by stage I & II (P = 0.609 and P = 0.363, respectively, Figure 3B and 3D). Additionally, after matching by molecular subtype, patients with IMPC had a significantly worse clinical outcome than patients with IDC (Figure 4).

### Univariate and multivariate analyses of prognostic factors in IMPCs

To estimate the clinical significance of various prognostic factors that may influence BCSS or RFS in IMPCs, we performed univariate and multivariate Cox regression analyses. As shown in Table 2, univariate analysis revealed that TNM stage, ER status, PR status, and HER-2 status were statistically significant prognostic factors for BCSS. Additionally, TNM stage, lymphovascular invasion, ER status, and PR status were statistically significant prognostic factors for the RFS of patients with IMPC (Table 2). In multivariate Cox proportional hazards regression analysis, we found that only TNM stage was an independent prognostic factor for BCSS (P = 0.031; HR = 4.217, 95% CI 1.141–15.579) and RFS (P = 0.005; HR = 3.554, 95% CI 1.467–8.609; Table 2).

**Table 2:** Univariate and multivariate survival analyses for BCSS and RFS.

Variables		BCSS				RFS			
		Univariate	Multivariate			Univariate	Multivariate		
		*P*	HR	95% CI	*P*	*P*	HR	95% CI	*P*
Age, year	≤50 vs.>50	0.356	1.911	0.768–4.755	0.164	0.582	1.047	0.554–1.982	0.887
TNM stage	Stage I & II vs. Stage III	**0.002**	**4.217**	**1.141–15.579**	**0.031**	**<0.001**	**3.554**	**1.467–8.609**	**0.005**
Lymphovascular invasion	Negative vs. Positive	0.060	1.920	0.397–9.277	0.417	**0.039**	1.151	0.398–3.331	0.795
ER status	Negative vs. Positive	**0.001**	0.493	0.126–1.920	0.308	**0.009**	0.748	0.269–2.082	0.578
PR status	Negative vs. Positive	**0.001**	0.560	0.195–1.610	0.282	**0.004**	0.644	0.283–1.465	0.294
HER-2 status	Negative vs. Positive	**0.045**	1.121	0.350–3.595	0.847	0.061	1.158	0.513–2.613	0.724

**Abbreviations**: BCSS, breast cancer-specific survival; RFS, recurrence/metastasis-free survival; HR, hazard ratio; CI, confidence interval; TNM, tumor, node, metastasis; ER, estrogen receptor; PR, progesterone receptor; HER-2, human epidermal growth factor receptor 2

**Bold values denote P<0.05.**

## Discussion

To the best of our knowledge, this study is the largest retrospective analysis of the clinico-pathological features of IMPC in a female Chinese population. IMPC is a rare pathological subtype of breast cancer, and the pure variant of IMPC is even more rare. In our study, the overall incidence of IMPC among primary invasive breast cancers was 1.8%. In this population, most patients (1.5%) had mixed IMPC, whereas only 0.3% had the pure form. The most common component of the mixed IMPC cases was IDC (77.1%), which is consistent with previous reports [5], [7], [10], [16]. In this study, we investigated the clinico-pathological features and prognosis of IMPC in comparison to IDC, which is the most common histological type of invasive breast cancer. In some respects, IMPC is quite similar to IDC. For instance, our study found no statistically significant differences between the two groups in terms of the age at diagnosis or menopausal status. However, there were more remarkable differences in the clinico-pathological characteristics and prognosis of IMPC and IDC. Several previous studies have reported that IMPC is an aggressive tumor with a poor clinical outcome [2], [3], [17]. Our study clearly demonstrated that the 5-year BCSS and RFS of patients with IMPC were significantly poorer than those of patients with IDC. IMPC tumors were significantly larger than IDC lesions in our study. One potential explanation could be that IMPCs are diagnosed at a larger size due to their relatively high proliferation rates. Previous studies have shown that IMPC is associated with lymphovascular invasion and a higher propensity for lymph node metastases [3], [6], [8], [17]. In our study, the incidence of lymphovascular invasion and axillary lymph node metastases in IMPC was 75.4% and 73.4%, respectively; these rates were significantly higher than those observed in IDC. The results of our univariate analysis also showed that both lymphovascular invasion and nodal involvement were important prognostic factors.

In this study, we detected no statistically significant differences in the 5-year BCSS and RFS rates between the IMPC group and the IDC group after matching by stage I & II. Interestingly, in a stage III-matched analysis, IMPC patients had significantly worse BCSS and RFS rates than IDC patients. Our multivariate analysis indicated that TNM stage was an independent prognostic factor for BCSS and RFS. Additionally, we found that stage III IMPC patients accounted for more than half of all patients with IMPC. Moreover, patients with IMPC had a significantly higher rate of stage III breast cancer than patients with IDC. This observation implies that IMPC tends to arise as a locally advanced disease, which is in accordance with a previous study [18]. Therefore, we speculated that these two points contribute to the lower BCSS and RFS rates of patients with IMPC compared to those of patients with IDC.

In the current analysis, the high percentages of ER and PR positivity in IMPCs (85.1% and 78.2%, respectively) are in accordance with other reports [10], [18], [19]. Generally, ER and PR positivity have been associated with a favorable outcome, and the results of our univariate analysis also showed that both ER and PR were favorable prognostic factors. We appear to have detected a paradoxical phenomenon in which the IMPC patients with higher percentages of hormone receptor positivity have worse clinical outcomes in comparison with IDC patients. Herein, we speculate that the unique histological characteristics of IMPC determine its more aggressive behavior and poorer outcome, in spite of its higher percentage of hormone receptor expression relative to IDC. Additional larger studies are warranted to confirm this finding.

Molecular subtype has been shown to be an important characteristic of breast cancer and is valuable in predicting the clinical outcome of IDC [20], [21], [22]. However, there is limited information specifically describing the molecular subtypes of IMPC. Upon investigation of the biological features of IMPC by molecular subtype, our data demonstrated that both the BCSS and RFS of patients with the luminal subtype were obviously better than those of patients with the non-luminal subtype. Paradoxically, the percentage of luminal subtype tumors was surprisingly higher in IMPC than in IDC. We further compared the survival of patients with IMPC and IDC stratified by molecular subtype. Stratification analysis indicated that both the BCSS and RFS of patients with IMPC were obviously inferior to those of IDC patients of any subtype. We therefore speculate that IMPC exhibits more aggressive behavior and a poorer outcome, regardless of its favorable molecular subtype, mainly due to its histological instinct. Further studies are necessary to elucidate this important issue.

We found that patients with IMPC were treated less frequently with breast-conserving surgery and were more likely to receive neoadjuvant chemotherapy and adjuvant radiotherapy than patients with IDC. These therapy choices are typically attributed to the relatively larger tumors, higher propensity of lymph node involvement and higher TNM stage associated with IMPC. Due to the increased rate of ER- and PR-positivity in IMPC, patients with IMPC were more likely to receive adjuvant endocrine therapy than those with IDCs. Chen et al. [17] also suggested that adjuvant endocrine therapy would improve the survival of patients with IMPC.

Some limitations of this study should be acknowledged. First, the retrospective nature of the data introduces bias. Second, the current study does not specify the proportion of the IMPC component in each lesion. However, no consensus has yet been reached on the proportion of the IMPC component in a tumor that is required for its pathological diagnosis [13]. In addition, previous studies did not find a direct correlation between the ratio of the IMPC component and either lymph node involvement or survival [9], [17]. Furthermore, pathology data addressing histological grade and Ki-67 were excluded from the current analysis, but these variables may have an effect on survival.

In conclusion, this study confirms that IMPC has a more aggressive behavior and a poorer outcome than IDC, regardless of the favorable molecular subtype. IMPC usually presents with a higher TNM stage. This higher TNM stage appears to be an independent adverse prognostic factor. Our findings illustrate that the poorer survival of patients with IMPC might be due to the increased incidence and aggressiveness of tumors in TNM stage III. These findings provide insight into the clinico-pathological characteristics and the prognostic factors of IMPC that will be of value for surgeons and pathologists.

## Supporting Information

Table S1 **Clinicopathological characteristics for pure IMPC patients and mixed IMPC patients.**
(DOC)
