# Supplementary material for: Clinico-Pathological Features and Prognosis of Invasive Micropapillary Carcinoma Compared to Invasive Ductal Carcinoma: A Population-Based Study from China
Source: PLoS One. 2014 Jun 30;9(6):e101390. doi: 10.1371/journal.pone.0101390 (PMC4076314; doi:10.1371/journal.pone.0101390)
Supplement: Table S1 — Clinicopathological characteristics for pure IMPC patients and mixed IMPC patients. (DOC) [file pone.0101390.s001.doc]

**SUPPLEMENTARY INFORMATION**

**Clinico-pathological Features and Prognosis of Invasive Micropapillary Carcinoma Compared to Invasive Ductal Carcinoma: A Population-based Study from China**

Wen-Biao Shi^1,2,3^, Lin-Jun Yang^3^, Xin Hu^1,2^, Jian Zhou^3^, Qiang Zhang^3^, Zhi-Ming Shao^1,2*^

**Authors' Affiliations**

1 Department of Breast Surgery, Key Laboratory of Breast Cancer in Shanghai，Fudan University Shanghai Cancer Center, Shanghai, China; 2 Department of Oncology, Shanghai Medical College, Fudan University, Shanghai, China; 3 Department of Surgical Oncology, Taizhou Municipal Hospital, Taizhou, Zhejiang, China.

Wen-Biao Shi, Lin-Jun Yang and Xin Hu contributed equally to this work.

**Competing Interests:** The authors have declared that no competing interests exist.

*** E-mail:** [zhimingshao@yahoo.com](mailto:zhimingshao@yahoo.com)

| **Table S1. Clinicopathological characteristics for pure IMPC patients and mixed IMPC patients.** | | | | | | | | | |
| --- | --- | --- | --- | --- | --- | --- | --- | --- | --- |
| **Characteristic** | |  | | **Pure IMPC** | | | **Mixed IMPC** | | ***P*^a^ Value** |
|  |  | |  | | **n=27** |  | **n=161** |  |  |
|  |  | |  | | **n** | **%** | **n** | **%** |  |
| **Age, year (Mean±SD)** | |  | | 50.6±11.8 | |  | 53.1±11.2 |  | 0.196 |
| **Menopausal status** | |  | |  | |  |  |  | 0.208 |
| Premenopausal | |  | | 15 | | 55.6 | 66 | 41.0 |  |
| Postmenopausal | |  | | 12 | | 44.4 | 95 | 59.0 |  |
| Unknown |  | |  | |  |  |  |  |  |
| **Tumor size, cm** | |  | |  | |  |  |  | 0.365 |
| T≤2 |  | |  | | 12 | 46.2 | 54 | 33.5 |  |
| 2＜T≤5 |  | |  | | 11 | 42.3 | 92 | 57.1 |  |
| T＞5 |  | |  | | 3 | 11.5 | 15 | 9.3 |  |
| Unknown |  | |  | | 1 |  |  |  |  |
| **Node status** | |  | |  | |  |  |  | 0.217 |
| 0 |  | |  | | 9 | 33.3 | 41 | 25.5 |  |
| 1—3 |  | |  | | 7 | 25.9 | 43 | 26.7 |  |
| 4—9 |  | |  | | 3 | 11.1 | 46 | 28.6 |  |
| ≥10 |  | |  | | 8 | 29.6 | 31 | 19.3 |  |
| Unknown |  | |  | |  |  |  |  |  |
| **TNM stage** |  | |  | |  |  |  |  | 0.346 |
| Ⅰ |  | |  | | 6 | 23.1 | 20 | 12.4 |  |
| Ⅱ |  | |  | | 8 | 30.8 | 57 | 35.4 |  |
| Ⅲ |  | |  | | 12 | 46.2 | 84 | 52.2 |  |
| Unknown |  | |  | | 1 |  |  |  |  |
| **ER status** |  | |  | |  |  |  |  | 0.563 |
| Positive |  | |  | | 22 | 81.5 | 138 | 85.7 |  |
| Negative |  | |  | | 5 | 18.5 | 23 | 14.3 |  |
| Unknown |  | |  | |  |  |  |  |  |
| **PR status** |  | |  | |  |  |  |  |  |
| Positive |  | |  | | 20 | 74.1 | 127 | 78.9 |  |
| Negative |  | |  | | 7 | 25.9 | 34 | 21.1 |  |
| Unknown |  | |  | |  |  |  |  |  |
| **HER2 status** | |  | |  | |  |  |  | 0.640 |
| Positive |  | |  | | 6 | 24.0 | 49 | 30.8 |  |
| Negative |  | |  | | 19 | 76.0 | 110 | 69.2 |  |
| Unknown |  | |  | |  |  |  |  |  |
| **Lymphovascular invasion** | |  | |  | |  |  |  | 0.329 |
| Yes |  | |  | | 21 | 84.0 | 114 | 74.0 |  |
| No |  | |  | | 4 | 16.0 | 40 | 26.0 |  |
| Unknown |  | |  | |  |  |  |  |  |
| **Abbreviations:** IMPC, invasive micropapillary carcinoma; IDC, invasive ductal carcinoma; TNM, tumor, node, metastasis; ER, estrogen receptor; PR, progesterone receptor; HER-2, human epidermal growth factor receptor 2 | | | | | | | | | |
| **P-value is caculated by two-sided χ2 test;** | | | | | | | | | |
| **Bold values denote P ＜ 0.05.** | | | | | | | | | |
